# An Account of an Abdominal Pregnancy

**Published:** 1813-10

**Authors:** John Ramsbotham

**Affiliations:** Old Jewry


### *An* ACCOUNT *of an* ABDOMINAL PREGNANCY.

IN the beginning of July last, Mrs. P. æt. 40, presented herself to the physician of a public dispensary for relief under a violent diarrhœa of long standing, attended with considerable pain in the abdomen, and general emaciation. She, however, did not then disclose any of the circumstances, hereafter to be detailed, which would have afforded a clue to the real cause and nature of her complaints. Some medicines were directed for her, and she was desired to attend again. A few days afterwards she died; and having always supposed supposed herself, contrary to the belief of her friends, to be pregnant, she had given orders that her body should be opened after death to ascertain the fact, or to determine the cause of her singular symptoms. Being in low circumstances, the body was removed to the poor-house of the parish in which she died, and the surgeon employed by the parish, who had never seen or heard of the case during life, was requested to inspect it. Upon opening the abdomen in the usual manner, there appeared in that cavity evident marks of long-continued inflammation; the peritonæum lining the abdominal muscles was closely and pretty generally adherent to the omentum and intestines, particularly at the lower part and on the left side; the omentum was altered from its natural appearance, was darker colored, and looser in its texture. From its general adhesion, the omentum could not be reflected upwards; but with little difficulty it was separated from the parts underneath, and this separation exposed a cavity containing numerous bones of a well-grown fœtus in a very putrid state, lying huddled together, but separate from each other, and intermixed with them a small quantity of an offensive dark-colored fluid. Even the bones of the head and of the pelvis were detached from each other. The bones being removed from their situation, and the cyst containing them cleaned out, it was found that the posterior parietes of the cyst were formed of the colon, mesentery, and neighboring parts; and that its anterior parietes were composed of the omentum, and the adhesions of the peritonæum lining the abdominal muscles. The extremity of the fallopian tube was lost in the general mass; and at that point where the cyst was united to the colon, absorption or destruction of a portion of that gut had taken place, so that an opening into the cyst, and out of it from the colon, was apparent. The size of the bones determined the fœtus to have reached the sixth or seventh month. The uterus itself was not in the least enlarged; the os uteri and the whole of its surface had a natural healthy appearance, as had likewise the right fallopian tube and ovaria.

From the husband of the deceased, and different individuals of her acquaintance, some interesting information has been obtained. This poor woman supposed herself to have conceived about the latter end of March or beginning of April 1812, for till near this time her catamenia had been regular, and she had been in good health; but after this time menstruation had disappeared. About nine years ago she bore a living child, and in the intermediate time had had two miscarriages. In the early part of this pregnancy she suffered greatly from sickness at stomach, violent pains in the

 belly, belly, and particularly from confinement of bowels; and after a time she found herself gradually increasing in size, chiefly on the left side of the belly. Towards the end of June, when she supposed herself nearly three months gone with child, she applied to a professional man for relief. At this time she complained of violent pain on the left side of the abdomen, striking through to the back; of sickness at stomach, and pain in the head; and obstinate confinement of bowels, upon which the strongest purgatives were found to have little effect. Even now there was a considerable tumor on the left side of the body. She told this gentleman she was positive she was with child, but that she was very different in her own feelings from the pregnancy preceding. The above symptoms were so violent as almost constantly to confine her to her bed for some weeks, and even to lead to a suspicion that she would miscarry. After a few weeks, however, she found herself somewhat better, and began to feel the motion of the child distinctly. This distinct motion was observed for six weeks or two months, when it ceased altogether.

A circumstance must now be mentioned to which she and her husband imputed many of her sufferings, but which a professional man will consider of little importance. Early in the month of June she was bit by a dog: this excited considerable alarm in her mind at the time, and the impression of her having sustained material injury from that accident remained to the day of her death.

In the month of August she removed from the neighbourhood in which she had lived, and bespoke a respectable midwife to attend her in her expected lying-in; and not long afterwards she sent for the midwife, supposing she was about to fall into labour, though not at her full time, in consequence of violent pains in the belly. These pains were so different from labour-pains, that the midwife told her she was not in labour. The midwife saw her several times under the same circumstances. For the relief of these pains, in September, another professional man was consulted. He found her complaining of violent pain on the left side of the belly, which was increased on pressure; the abdomen enlarged to the size of the fifth or sixth month of pregnancy; obstinate costiveness; and a disposition to general emaciation. For the relief of these symptoms, opening medicines and opiates were prescribed.

During the months of October and November, the midwife was repeatedly sent for in consequence of this poor woman having pains not unlike labour-pains, with a sensation of bearing down, and an occasional colored discharge from from the vagina. There seemed, however, no disposition in the parts for labour, so that the midwife at length gave it as her opinion that the woman was not with child. About this time a prolapsus of the vagina took place.

In the beginning of December, she was again visited by the same professional gentleman, who found her under occasional pains not unlike labour-pains, with a colored discharge, and a sense of bearing down. On examination, he observed something external to the parts, which proved to be a prolapsus of the vagina: there was, however, no disposition to labour. The belly was considerably swelled and hard; the bowels confined: still the poor woman expressed a confident belief that she was pregnant and at her full time, but from the difference in her sensations, not having felt motion for some time past, she suspected the child to be dead.

These symptoms continued more or less till the beginning of February 1813, when a violent diarrhœa commenced, attended with pain, and further emaciation of body. Her evacuations in the early part of it were particularly offensive, numerous, and large; and pieces of putrid animal substances were observed among them; and at one time she passed by stool one of the ossa femoris. She now expressed a belief that she was passing the dead child by stool. From the time that the diarrhœa took place, her belly began to diminish in size, and the rest of her body to waste away.

These symptoms continued more or less to the time of her death in the following July; but she was able to crawl about in the open air till within a few days of that event, and to take nourishment.*

*Old Jewry,* JOHN RAMSBOTHAM, M.D.

*Sept.* 6, 1813.

---

*To the Editors of the Medical and Physical Journal.*

GENTLEMEN,

THE following case having come recently under my care, I have taken the liberty to transmit you the particulars, not so much as possessing peculiar claims to your attention, as to prove the decided efficacy of medicine.

---

* It is to be regretted that Dr. Ramsbotham did not attend this patient during her life. As it is, we are indebted to him for the relation of such particulars as he could collect; and we know, that he took great pains to obtain information from every individual who could throw light upon the subject. If any inaccuracy in the statement has occurred, allowance must be made, especially too, as it is entirely owing to the exertions of Dr. R. that the case is recorded.—EDITORS.

